# Supplementary material for: Diffusional Kurtosis Imaging of White Matter Degeneration in Glaucoma
Source: J Clin Med. 2020 Sep 27;9(10):3122. doi: 10.3390/jcm9103122 (PMC7600134; doi:10.3390/jcm9103122)
Supplement: Supplementary file 1 [file jcm-09-03122-s001.pdf]

Table S1. Localization of brain regions which present statistically significant differences in fractional anisotropy (FA) (control (CTRL) > glaucoma group (GLA)). MNI: Montreal Neurological Institute. White Matter (WM).

WM region	Cluster size (voxels)	1-*p* (max)	MNI X (mm)	MNI Y (mm)	MNI Z (mm)
Body corpus callosum	1243	0.974	−5	19	16
Genu corpus callosum	1030	0.978	11	32	−1
Splenium corpus callosum	87	0.956	9	−33	25
Left-Anterior corona radiata	138	0.978	−16	29	25
Right-Anterior corona radiata	140	0.976	15	34	−5
Left-Superior corona radiata	73	0.972	−17	15	31
Forceps minor	3607	0.978	11	32	−1

Table S2. Localizations of brain regions which present statistically significant differences in kurtosis anisotropy (KA) (CTRL) > glaucoma group (GLA)). MNI: Montreal Neurological Institute. White Matter (WM); optic radiation (OR), inferior longitudinal fasciculus (ILF), inferior front-occipital fasciculus (IFOF), superior longitudinal fascicle (SLF).

WM region	Cluster size (voxels)	1-*p* (max)	MNI X (mm)	MNI Y (mm)	MNI Z (mm)
Body corpus callosum	1652	0.972	−6	−10	26
Genu corpus callosum	967	0.974	10	33	1
Splenium corpus callosum	1257	0.972	12	−41	11
Left-Posterior thalamic radiation (OR)	99	0.972	−26	−63	12
Right-Posterior thalamic radiation (OR)	106	0.971	30	−56	16
Left-Sagittal stratum (ILF/IFOF)	15	0.95	−38	−22	−7
	13	0.954	−38	−10	−16
Left-SLF	193	0.962	−36	−43	21
	136	0.956	−36	−10	25
Left-Anterior corona radiata	130	0.957	−25	14	26
	126	0.968	−16	35	6
	118	0.962	−17	25	23
Right-Anterior corona radiata	164	0.973	15	34	−5
Left-Superior corona radiata	68	0.957	−27	10	28
	58	0.964	−18	2	39
	40	0.963	−21	−29	39
Left-Anterior limb internal capsule	68	0.952	−10	3	−3
Right-Cingulate gyrus (hippocampus)	93	0.963	24	−34	−9
Left-Cingulate gyrus (cingulum)	21	0.971	−7	10	29
	12	0.955	−8	−24	32
	10	0.972	−7	−13	33
Left-Uncinate fasciculus	13	0.954	−34	−1	−21
Forceps major	1482	0.972	12	−41	11
Forceps minor	2503	0.974	10	33	1
Left-Fusiform Gyrus (Occipital lobe) WM	57	0.951	−32	−57	−8
Left-Fusiform Gyrus (Temporal lobe) WM	8	0.954	−42	−9	−28
	3	0.951	−50	−39	−15
	3	0.951	−48	−45	−12

Table S3. Localization of Brain regions which present statistically significant differences in mean kurtosis (MK) (CTRL) > glaucoma group (GLA)). MNI: Montreal Neurological Institute. White Matter (WM); optic radiation (OR), inferior longitudinal fasciculus (ILF), inferior front-occipital fasciculus (IFOF), superior longitudinal fascicle (SLF).

WM region	Cluster size (Voxel's number)	1-*p*	MNI X (mm)	MNI Y (mm)	MNI Z (mm)
Body corpus callosum	2114	0.985	3	19	15
Genu corpus callosum	1349	0.985	12	33	−1
Splenium corpus callosum	1511	0.984	16	−42	10
Left-Posterior thalamic radiation (OR)	449	0.984	−30	−69	11
Right-Posterior thalamic radiation (OR)	358	0.984	29	−64	13
Left-Sagittal stratum (ILF/IFOF)	176	0.982	−38	−9	−17
	130	0.973	−36	−50	−8
Right-Sagittal stratum (ILF/IFOF)	106	0.977	44	−35	−8
	100	0.978	39	−9	−18

Left-SLF	1302	0.985	−36	−43	20
	153	0.984	−33	0	21
Left-Anterior corona radiata	422	0.985	−16	26	21
	332	0.984	−25	27	13
Right-Anterior corona radiata	1104	0.985	16	37	3
Left-Superior corona radiata	774	0.985	−18	−7	41
	100	0.985	−17	9	34
Left-Anterior limb internal capsule	553	0.981	−11	3	−3
Right-Anterior limb internal capsule	327	0.978	23	22	1
Right-Posterior limb internal capsule	516	0.975	15	−6	2
Left-Cingulate gyrus (cingulum)	69	0.985	−7	10	28
	24	0.984	−8	−25	32
Right-Cingulate gyrus (hippocampus)	39	0.983	23	−40	−4
Left-Corticospinal tract	153	0.972	−4	−27	−34
Middle cerebellar peduncle	718	0.972	−14	−30	−30
Left-Uncinate fasciculus	33	0.981	−36	−5	−15
Forceps major	3069	0.985	−30	−59	24
Forceps minor	4571	0.985	12	33	−1
Left−Fusiform Gyrus (Occipital lobe) WM	187	0.973	−33	−57	−11
Left-Fusiform Gyrus (Temporal lobe) WM	80	0.973	−34	−46	−12
Right-Fusiform Gyrus (Occipital lobe) WM	55	0.966	31	−57	−11
Right-Fusiform Gyrus (Temporal lobe) WM	37	0.966	37	−39	−16
	18	0.966	33	−43	−15

Table S4. Brain regions which present statistically significant differences in radial kurtosis (RK) (CTRL) > glaucoma group (GLA)). MNI: Montreal Neurological Institute. White Matter (WM); optic radiation (OR), inferior longitudinal fasciculus (ILF), inferior front-occipital fasciculus (IFOF), superior longitudinal fascicle (SLF).

WM region	Cluster size (Voxel's number)	1-*p*	MNI X (mm)	MNI Y (mm)	MNI Z (mm)
Body corpus callosum	2065	0.987	3	17	16
Genu corpus callosum	1239	0.987	10	33	3
Splenium corpus callosum	633	0.974	14	−43	13
	325	0.979	−6	−40	18
	243	0.98	−25	−53	18
Left-Posterior thalamic radiation (OR)	276	0.98	−27	−70	8
	136	0.969	−37	−47	−3
Right-Posterior thalamic radiation (OR)	296	0.974	30	−57	15
Left-Sagittal stratum (ILF/IFOF)	150	0.979	−38	−9	−17
	74	0.978	−42	−39	−7
Right-Sagittal stratum (ILF/IFOF)	94	0.967	41	−14	−14
	24	0.966	42	−36	−9
Left-SLF	1067	0.985	−31	10	25
Left-Anterior corona radiata	428	0.986	−17	24	26
	266	0.985	−17	28	−12
Right-Anterior corona radiata	548	0.987	15	33	−8
	186	0.979	26	14	24
Left-Superior corona radiata	226	0.978	−27	−16	23
	165	0.985	−25	11	25
Left-Anterior limb internal capsule	487	0.984	−21	14	12
Right-Anterior limb internal capsule	302	0.979	20	20	0
Right-Posterior limb internal capsule	375	0.979	24	−21	3
Left-Cingulate gyrus (cingulum)	48	0.984	−7	5	30
Middle cerebellar peduncle	176	0.954	−32	−53	−41
Left-Uncinate fasciculus	20	0.965	−34	1	−12
Forceps major	1140	0.974	14	−43	13
	1050	0.98	−28	−51	16
Forceps minor	4646	0.987	9	33	2
Left-Fusiform Gyrus (Occipital lobe) WM	133	0.973	−27	−61	−9
Left-Fusiform Gyrus (Temporal lobe) WM	66	0.966	−32	−42	−16
